# Clinical evaluation of single and repeated sessions of photobiomodulation with two different therapeutic wavelengths for reducing postoperative sequelae after impacted mandibular third molar surgery: a randomized, double-blind clinical study

**DOI:** 10.1590/1678-7757-2021-0383

**Published:** 2021-11-15

**Authors:** Mehmet Nuri YÜKSEK, Cennet Neslihan EROĞLU

**Affiliations:** 1 Yuzuncu Yil University Faculty of Dentistry Department of Oral and Maxillofacial Surgery Van Turkey Yuzuncu Yil University, Faculty of Dentistry, Department of Oral and Maxillofacial Surgery, Van, Turkey.

**Keywords:** Low-level laser therapy, Laser therapy, Photobiomodulation therapy, Third molar

## Abstract

The effects of the number of photobiomodulation (PBM) sessions on the postoperative inflammatory process remain controversial. The current literature comparing single and repeated PBM sessions is limited.

**Objective:**

This study aimed to compare the effects of single and repeated PBM sessions, applied at two different therapeutic wavelengths within the infrared spectrum, on postoperative inflammatory response after impacted third molar tooth extraction.

**Methodology:**

This randomized, double-blind clinical study included 40 patients with bilateral impacted mandibular third molars (80 teeth). The patients were divided into two groups each including 20 subjects (40 teeth) to receive either single-session laser at 810 nm (20 teeth) and 940 nm (20 teeth) immediately after the surgery or repeated laser sessions at 810 nm (20 teeth) and 940 nm (20 teeth) (immediately after the surgery and on postoperative Day 1). Lasers at 940 nm (power density 0.5 Watt/cm2, energy density 4 J/cm2 for a time until the cumulative energy on the device screen reaches 50 J from 0 J, in continuous mode, spot size 2.8 cm2) and at 810 nm (power density 0.14 Watt/cm2, energy density 4 J/cm2, for 30 seconds, in continuous mode, spot size 2.1 cm2) were applied intra- and extra-orally. Pain, swelling, and trismus were evaluated postoperatively.

**Results:**

No significant differences were determined between the groups on the evaluated parameters (p>0.05).

**Conclusion:**

Within the study limitations, in PBM, the effects of 810 nm and 940 nm and those of single and repeated applications were similar regarding pain, swelling and trismus. Immediate postoperative PBM could be preferred to repeated applications performed by point application within a 24-hour period.

## Introduction

The inflammatory process following impacted tooth extraction is a clinical event during which the effects of many anti-inflammatory agents are evaluated, such as in bone and soft tissue surgeries and related complications. Pain, swelling, and trismus hinder the quality of life of patients and make the postoperative period challenging. Various medical agents and atraumatic surgical methods have been tried to suppress these effects. However, since the medical agents used to provide a solution for postoperative problems have potential side effects, clinicians turned their attention to photobiomodulation (PBM) therapy (PBMT), considered to have no side effects.^1 - 3^ Currently, PBM is defined as “ *the mechanism through which nonionizing optical radiation in the visible and near-infrared spectral range is absorbed by endogenous chromophores to elicit photo-physical and photo-chemical events at various biological scales without eliciting thermal damage* ”.^4^ Accordingly, the term PBM is recently used to enlarge the effect of biostimulation not only to low-level lasers but also to all nonionizing light sources in the visible and near-infrared spectrum including lasers, light-emitting diodes, and broadband light and the term PBMT is defined as photon therapy based on the principle of PBM.^4^ While some authors defend that PBMT has no effect on pain, swelling, and trismus following impacted tooth extraction, others cite it as an effective and useful method for inflammation relief.^5 - 7^

Recently, the effects of the number of PBM sessions on the postoperative inflammatory process have become one of the controversial topics; most studies used repeated sessions.^8 - 12^The time spent for clinical visits is the major disadvantage for both patients and clinicians when considering repeated applications. Recent studies have focused on whether single-session application would provide the same outcome as the one achieved by repeated sessions.^13 , 14^ Nevertheless, to the best of our knowledge, the number of studies currently available comparing single and repeated sessions of PBM is limited to one.^15^

Laser wavelength is one of the factors altering the effects on the target tissue. The therapeutic wavelength range for low-level lasers is between 630 nm and 980 nm.^16^ Wavelengths between 600-700 nm are preferred for superficial tissues; wavelengths between 780-950 nm can achieve deeper optical penetration and are preferred for the treatment of deep tissues. Wavelengths between 700-770 nm are not considered sufficiently active.^17^ To the best of our knowledge, no comparative study showing the extent of benefit provided by lasers applied at between 780-950 nm wavelengths in therapeutic range for the treatment of deep tissues exist. Most studies in the literature were performed using 810-830 nm lasers.^6^

Thus, the present randomized, double-blind clinical study aimed to compare the efficacy of single-session and repeated PBM sessions applied using low-level lasers at two different wavelengths, both within the therapeutic range for deep tissues, on pain, swelling, and trismus following impacted third molar tooth extraction.

## Methodology

### Human subjects

**Figure 1:**
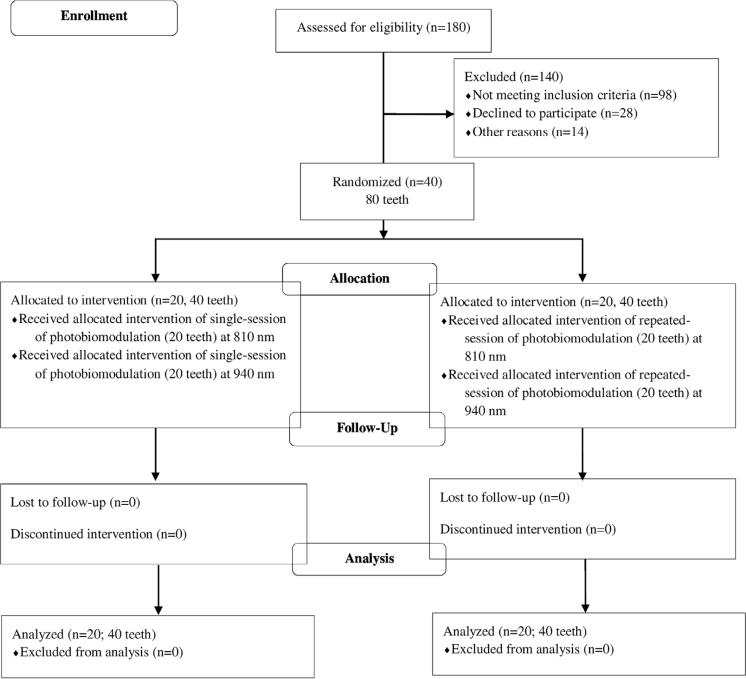
Patient flow diagram

All interventions and data acquisition performed in the present study were conducted in accordance with the ethical standards and approved by the Clinical Research Ethics Committee of Medical School of Van Yuzuncu Yil University with the approval no. 07032017.09. All procedures were performed in accordance with the 1964 Helsinki Declaration and its later amendments. Written informed consent of all patients were obtained after informing them about the study objectives and methods. The quality assessment was conducted according to the CONSORT Statement RCT checklist and Figure 1 shows the patient flow diagram. This study was retrospectively registered in Thai Clinical Trials Registration with the trial registration number TCTR20200818007.

### Inclusion and exclusion criteria

In total, 180 patients, who were admitted to the department of oral and maxillofacial surgery in Van Yuzuncu Yil University, Faculty of Dentistry, Van, Turkey, were examined within a 3-month period and were indicated for removal of impacted mandibular third molars. The teeth were duly classified as Class 2 and Class B according to the Pell-Gregory classification and to be in the mesioangular position according to the Winter’s classification. Exclusion criteria included patients with systemic diseases, on medication within the last month, pregnant or lactating women, history of allergy against the medications likely to be prescribed in the postoperative period, and apparent asymmetry between the left and right mandibular third molars. Patients who developed postoperative alveolitis and who did not attend the control visits were also excluded. Patients with recent or current infection or lymphadenopathy were also excluded. Thus, 40 eligible patients aged between 18-36 years, who had bilateral impacted mandibular third molars with bone retention and indication for surgical extraction, were included in this study.

### Randomization and group determination

The patients (n=40, 80 teeth) were randomly divided into two groups with 20 patients (40 teeth) to receive either single-session laser at 810 nm (20 teeth) and 940 nm (20 teeth) or repeated session laser at 810 nm (20 teeth) and 940 nm (20 teeth). Group allocation (either single or repeated session group) to which a patient would be included was decided by tossing a coin^18^ while setting their appointments for the surgery. The day each patient was admitted for the first tooth removal, the side (right or left) that would be operated first and the wavelength (940 nm or 810 nm) to be applied were also decided by coin toss in both groups. PBM was applied immediately after the extraction (Day 0) in the single-session group and immediately after the extraction (Day 0) and one day after the extraction (Day 1) in the repeated session group.

### Blinding

The surgeon was blinded to the groups, laser devices to be used, and the number of laser sessions to be applied. The physician who performed the laser applications was not blinded. The patients had no information about the laser devices and the number of laser sessions.

### Surgical procedure

The same surgeon (MNY) performed all operations. The surgeon had over 5 years of experience in maxillofacial surgery. Prior to the operation, the surgeon was only informed about the side to be removed first for each patient according to the results of the coin toss. During the surgical procedure, first, inferior alveolar and buccal nerve block was performed using a local anesthetic solution composed of articaine hydrochloride (40mg/mL)+epinephrine (0.012 mg/mL). The procedures were performed with an auxiliary incision at 45º from the mouth to the vestibule following the gingival incision beginning from the anterior sharp edge of the mandibular ramus (margo anterior rami mandibulae) to the mandibular second molar tooth. Retentive bone was removed by standard procedures and the tooth was extracted by fragmentation. To avoid extra heat and trauma in the region, the drills for osteotomy were only used once for each tooth. In all patients, the extraction cavity was irrigated with 20 mL of sterile saline solution and then primarily closed using a 4/0 silk suture. Surgical duration from the initial incision to the last suture was estimated in the single and repeated session groups for both 810 nm and 940 nm laser applications.

In the postoperative period, all patients were prescribed with 1000 mg amoxicillin twice a day for 5 days, starting immediately after the surgery as the routine antibiotic prophylaxis, with benzydamine HCL+chlorhexidine gluconate as mouthwash three times a day for 5 days started immediately after surgery, and with 100 mg flurbiprofen twice a day for 3 days. Paracetamol 500 mg tablet was given as rescue analgesic. Patients were told to record the time they took the medication and the number of tablets, and instructed on how often they could take the medication. The surgery on the opposite side was performed using the same procedures with an interval of at least 3 weeks. The surgical procedures were performed at the same hours if possible – in the morning on Monday, Tuesday, and Wednesday – and by the same team.

### Laser application

The Biolase Ezlase 940 diode laser (Biolase Ezlase^®^ 940 Class IV; Biolase Technology, Inc., Irvine, CA) was used for laser application. The device was used in continuous mode and the application was performed both extra-orally (noncontact mode; by regional application) and intra-orally (noncontact mode) using the following laser parameters: 2.8 cm^2^ spot size, approximately 20 seconds in exposure duration (time elapsed until the cumulative amount of energy on the device screen went from 0 to 50 J), 4 J/cm^2^ energy density, 0.5 mW/cm^2^ power density. A bleaching handpiece (Biolase Technology, Inc., Irvine, CA) was also used. The handpiece used for the 940 nm laser device is the regional application handpiece that has a rectangular application surface recommended by the manufacturer for PBM. After placing the laser probe onto the skin, the energy was delivered to a triangular region that is bordered by the masseter muscle insertion in the angulus mandible, auricular tragus lobe, and the mesial margin of the mandibular second molar. Intra-oral irradiation was performed using the non-contact mode in-between the buccal wall of the extraction socket and the sulcus targeting the masseter mass, the area where swelling was expected to occur.

An 810-nm diode laser device (Cheese Dental Diode Laser^®^ DEN4A Class IV, Lotus Global Co., Ltd., London, UK) was used for laser application at 810 nm. The device was used in continuous mode and the application was performed both extra-orally (contact mode; by point application) and intra-orally (noncontact mode) using the following laser parameters: 2.1 cm^2^ spot size, 30 seconds exposure duration, 4 J/cm^2^ energy density, 0.14 mW/cm^2^ power density. The handpiece (Lotus Global Co., Ltd., London, UK) used for the 810 nm laser device is one of the handpieces recommended by the manufacturer for PBM because it has circular diameter and provides point application. The extra-oral irradiation was performed to the mandibular angulus, where the masseter muscle is attached to, and intra-oral irradiation was performed in-between the buccal wall of the extraction socket and the sulcus targeting the masseter mass, the area where swelling is expected to occur.

### Postoperative assessment of pain, swelling, and trismus

The patients were evaluated postoperatively for pain, swelling, and trismus by the surgeon. Pain was assessed using the Visual Analog Scale (VAS), which is a straight line with the digits from 0 on one end to 10 on the other end. On this 10-cm straight line, 0 represents no pain and 10 represents unbearable pain. After the operation the patients were asked to assess the intensity of pain and mark on the scale on the forms provided at the 1^st^, 2^nd^, 3^rd^, and 6^th^ postoperative hours and on postoperative days 1, 2, 3, 5, and 7. With these forms, the intensity of pain experienced by patients within one-week until the removal of sutures was evaluated.^19^

For the assessment of postoperative swelling, the distances between certain anatomical points on the face were measured with a flexible ruler before the surgery on postoperative days 2 and 7. For this purpose, the distances between three different points (tragus-oral commissure, tragus-soft tissue pogonion) were measured and recorded. The arithmetical mean of these distances was recorded as the facial distance (in millimeters).^13 , 20^ Trismus was evaluated by measuring the maximal inter-incisal distance both before the surgery (Day 0) and on the postoperative Days 2 and 7 using a digital caliper (Mitutoyo Corp., Japan) and the outcomes were recorded as the mouth opening (in millimeters).

### Statistical analysis

Data analyses were performed using the IBM SPSS Statistics for Windows version 23 (IBM Corp., Armonk, NY, USA). The differences between the groups were analyzed by the Mann-Whitney U test. All evaluations were performed within a 95% confidence interval and a p<0.05 was considered statistically significant.

## Results

This study included 40 patients (28 females and 12 males) and mean age was 21.5 years (range 18-36 years); 80 impacted teeth extractions were performed. The mean duration of surgery (from initial incision to the last suture) in the single session group was 14.3 and 14.1 minutes in the 810 nm and 940 nm groups, respectively. In the repeated session group, the mean duration of surgery was 13.8 and 14.5 minutes in the 810 nm and 940 nm groups, respectively.

No problem was found during the recovery period in patients with complete data. The patients used a similar number of analgesic tablets, and rescue analgesic was unnecessary.

**Figure 2:**
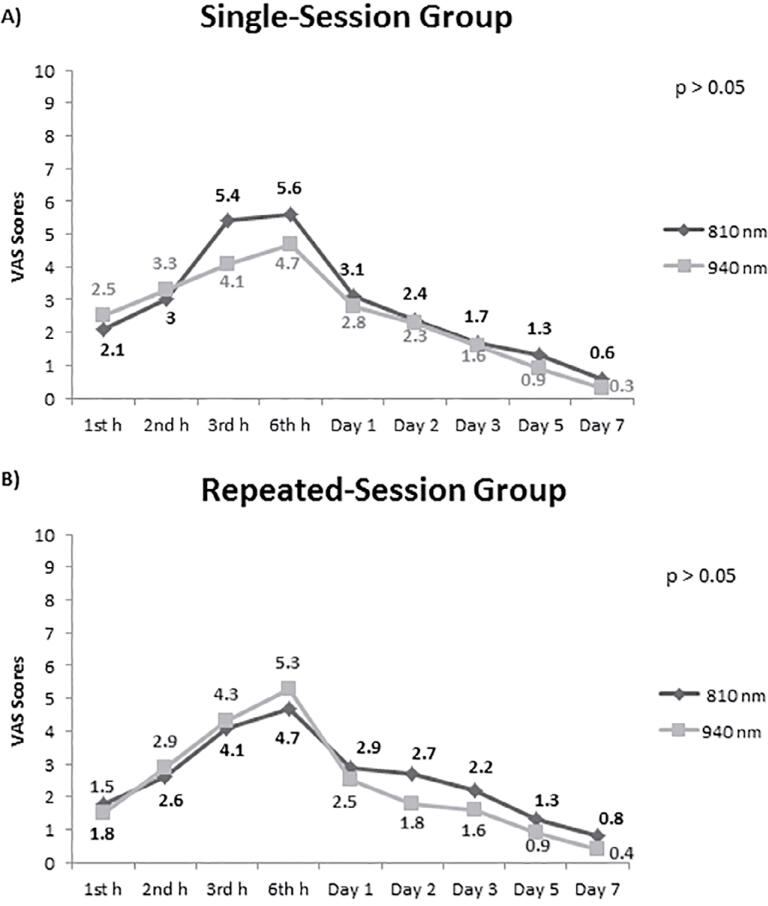
Comparison of the 810 nm and 940 nm groups in terms of pain severity at measuring time points in single-session and repeated session laser groups

Both in the single-session and repeated session groups, no significant difference was determined between the 810 nm and 940 nm groups in terms of the mean VAS scores at each measuring time point (p>0.05 for all; Figure 2 ).

**Figure 3:**
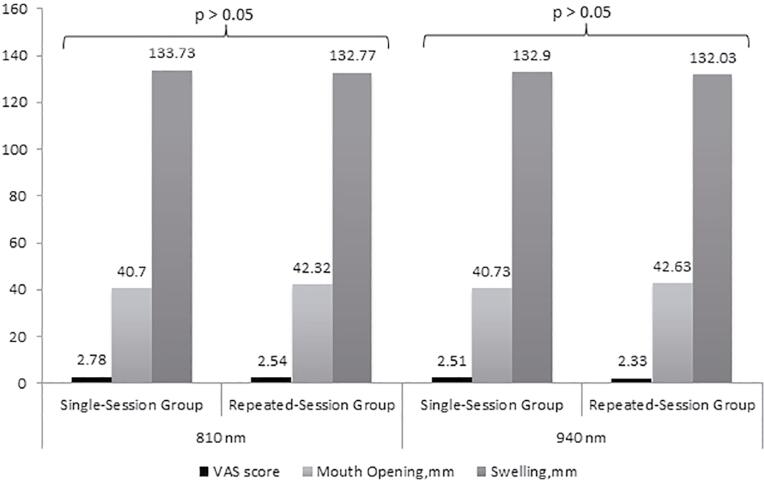
Results of the comparisons of the single- and repeated session groups in terms of visual analog scale scores, maximum mouth opening, and degrees of swelling for application at 810 nm and at 940 nm

There was no significant difference between the single-session and repeated session groups in terms of the mean VAS scores (p>0.05 for application at 810 nm and p>0.05 for application at 940 nm; Figure 3 ).

**Figure 4:**
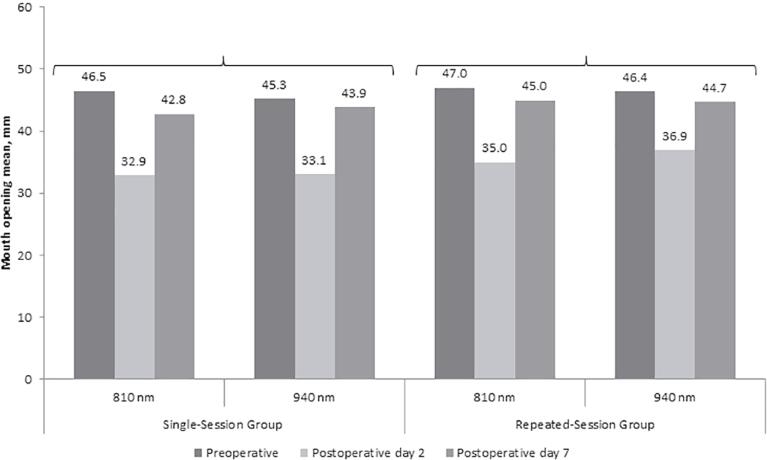
Comparisons of the 810 nm and 940 nm groups in terms of mouth opening in single-session and repeated session laser groups

On the postoperative Day 2 and Day 7, we did not observe significant difference between the 810 nm and 940 nm groups in terms of mean maximum mouth opening measured before the surgery (p>0.05 for each; Figure 4 ), both for the single-session and repeated session groups. Also, we did not find significant difference between the single-session and repeated session groups in terms of maximum mouth opening (p>0.05 for application at 810 nm and p>0.05 for application at 940 nm; Figure 3 ).

**Figure 5:**
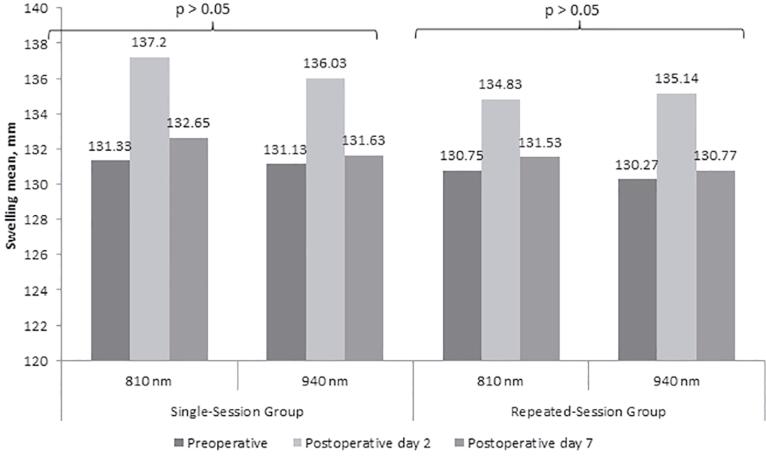
Comparisons of the 810 nm and 940 nm groups in terms of swelling in single-session and repeated session laser groups

No significant differences was observed between the 810 nm and 940 nm groups in terms of the mean degree of swelling measured on the postoperative Day 2 and Day 7 both for the single-session and repeated session groups (p>0.05 for all; Figure 5 ). No significant difference was determined between the single-session and repeated session groups in terms of the mean degree of swelling (p>0.05 for application at 810 nm and p>0.05 for application at 940 nm; Figure 3 ).

## Discussion

Laser therapy is still expensive when compared to other methods used to reduce postoperative pain, edema, and trismus.^21^ The cost of a laser device depends on the operating wavelengths because devices operating at high wavelengths have higher costs. Nevertheless, an appropriate wavelength within the therapeutic range should be used in order to obtain a clinically effective outcome. However, in clinical practice, it is unclear if different wavelengths within the therapeutic range cause difference. Another unclear issue is if repeated sessions are necessary for PBM to decrease postoperative inflammatory sequelae. This study evaluated these two issues and thereby investigated if PBM application could become more time- and cost-effective.

Level of penetration changes depending on the structure of the target tissue and the wavelength of the laser.^22^ To the best of our knowledge, this is the first study that compared the effects of different wavelengths within the therapeutic spectrum; therefore, we believe that this study will assist future studies.

Despite the unlikely symmetry due to tissue characteristics, split-mouth technique provided similar penetration depths for both sides, since the laser beam in the same wavelength pass in the same distance through the tissue. This represents an advantage of our study. Sierra, et al.^23^ (20116) compared the extra-oral and intra-oral laser applications in two different wavelengths (660 nm and 808 nm) within the red and infrared spectrum and found that extra-oral application was associated with better outcomes at 808 nm than that at 660 nm, although the difference was not statistically significant. Another study reported that there was an increase in local circulation in the groups that received photobiomodulation with a wavelength of 808 nm compared to the groups that did not receive any laser. Increased circulation had an effect in favor of the laser groups in terms of postoperative pain, swelling, and trismus, but it was not statistically reflected.^24^ Since the complications following impacted tooth extraction influence the subcutaneous tissues more intensely (reactions beginning from the deep tissues ending in the subcutaneous tissues), penetration of 600-700 nm within the red spectrum might have remained superficial and insufficient.^17^ For this reason, in PBMT performed for preventing the complications after impacted tooth extraction, taking the range for the treatment of deep tissues as the therapeutic range and using the wavelengths within this therapeutic range for optical penetration distance may be more convenient.^25^ Nevertheless, in this study, no significant differences were determined between the effects of the wavelengths (810 nm and 940 nm) in the infrared spectrum in terms of pain, edema, and trismus (Figures 2, 4, and 5). The differences between the laser spot sizes made the standardization of extra-wavelength parameters difficult. The actual energy density given to the target tissue and energy distribution in the tissue is unclear since the energy density increases with decreasing laser spot size.^25^ Although the energy density was taken as 4 J/cm^2^ based on earlier studies,^6 , 12 - 14 , 26^ there might have been spot size-related differences in the actual energy density that reached the target tissue. Moreover, using the probe in contact or in non-contact mode is another factor that could lead to a difference in energy density. The differences in spot sizes of laser devices were originated from using appliance tips according to the manufacturers’ instructions. In this study, we tried to keep the spot size close for both laser devices using the contact and non-contact modes.

Although it has been defended that penetration depth and anti-inflammatory effect of laser decreases with increasing wavelength,^27 - 29^ in this study, no significant difference was determined between the 810 nm and 940 nm wavelengths in terms of their clinical efficacy. Despite pain, swelling, and trismus values in the applications with 810 nm wavelength are expected to be lower than the values in the applications with 940 nm; when the data presented in Figure 2 is examined, we can observe that they are at very close levels, regardless of the single or double session application. In photobiomodulation, besides the depth of penetration of the wavelength of the laser, the effects on the local circulation may not be the same as well.^24^ Therefore, the anti-inflammatory effect may be different for each wavelength and each tissue.^29^ In the literature, studies demonstrated that a laser beam at 880 nm penetrated 54% deeper than a laser beam at 940 nm in bovine tissue samples.^27^ Brosseau, et al.^29^(2000) also found that laser at 632 nm wavelength was more effective on pain as compared with laser at 820 nm wavelength. Apart from these, we did not find any study reporting the wavelength penetration and anti-inflammatory effects in clinical detail.

Given that pain reaches the maximum level within 3-5 hours after local anesthesia and that edema and trismus reach the maximum degree 12-48 hours later, repeated applications target to prevent or reduce postoperative complications using applications performed in the postoperative first two days and to provide earlier resolution of the inflammatory response using applications performed after the 2^nd^ day.^8 , 9 , 14 , 22^ Three to four sessions per week could not be used as a method to prevent/decrease inflammation. However, a few more sessions can be added after the first 48 hours to provide faster resolution of inflammation or a few PBM applications can be performed within the first 48 hours; that is, within the same day; to see if different outcomes could be achieved. Nevertheless, it should be considered that such applications could be seriously time-consuming both for patients and for clinicians and would be far from practical. The literature demonstrated that immediate applications (performed immediately after surgery and on the postoperative 3^rd^ day) are more effective when compared with late applications (performed on the postoperative 2^nd^ and 4^th^ days).^1^

In this study, if pain, edema, and trismus values at 1^st^ and 3^rd^days, 3^rd^and 7^th^days, and 1^st^ and 7^th^ days were compared, different results could be observed, because the postoperative complications increased in the first 48 hours and then gradually decreased. However, our study aim was to make an efficacy evaluation on reducing/preventing the occurrence of these complications.

Some researchers argued that irradiating the whole problematic region provides better healing as compared with point irradiation.^30^ In this study, performing point irradiation did not significantly differ from the irradiation of the whole problematic region in the contact and non-contact modes. da Cunha, et al.^31^ (2008) reported a penetration depth of 1-5 cm in soft tissue for a device operating at a wavelength of 830 nm. Additionally, the studies suggesting a decrease in penetration depth and the anti-inflammatory effect of laser with increasing wavelength have been already mentioned above.^27 - 29^ Therefore, no difference may occur in terms of anti-inflammatory effect between performing laser application in the contact mode with the 940 nm device and in the non-contact mode with the 810 nm device.

Whereas some studies could be given as examples of the studies demonstrating significant effects of repeated applications of PBM using low-level lasers on the postoperative complications,^8 - 10^ the other studies concluded that repeated applications of PBM using low-level lasers had no significant effect as compared with the controls.^11 , 12^ This also applies to the single-session applications. Whereas some authors stated that single-session laser application was adequate,^13 , 16 , 23 , 32 , 33^ several authors reported that cumulative effect provided by single-session laser application was not adequate.^26 , 34 , 35^ Standardization has been frequently articulated for the differences between the results of laser studies. Nevertheless, sample size also needs to be considered. The results of this study showed that single-session application was adequate, being consistent with other studies. Koparal, et al.^15^ (2018) conducted the closest study to this study and compared ice compression for the pain, edema, and trismus following impacted tooth extraction with single-session and repeated sessions of PBM and concluded that no significant difference was observed between the groups in terms of preventing or reducing the inflammatory response. In this study, we also concluded that repeated applications of PBM resulted in no difference; however, an increase in the number of patients may change the outcome.

The magnitude of complications observed in the postoperative period is related to the inflammatory process that started as a result of surgical trauma in impacted tooth extractions and is expected to occur. However, the inexperience of the surgeon, the prolongation of the operation time, the surgery performed around the masseter, the sharpness of the instruments used, the errors during incision and dissection, anesthesia error, non-compliance with sterilization, etc. causes more surgical trauma. Therefore, the inflammatory response of the body increases. As a result, postoperative pain, swelling, and trismus may reach higher than expected values.^36^ In our study, no situation could cause extra surgical trauma mentioned above.

Evaluation of the findings of trismus and edema suggested that the methods of measurements used in this study might affect the outcomes. Measurement of distances through magnetic resonance imaging or ultrasonography while evaluating edema may have resulted in different outcomes.^37 , 38^ The findings related to trismus might have been affected by pain. We considered that this issue is a limitation in many studies addressing the models of impacted mandibular third molars and all groups.

Among the study limitations, single-session applications have significant advantages both for patients and for clinicians (such as reduced time loss for both, less clinical workload, joining social environment not being compulsory for the patients while coming to the hospital for a clinical visit during the healing process). Moreover, the absence of an extra clinical visit within the healing period is convenient. Therefore, the trend has recently been shifting to single-session laser applications. Abdel-Alim, et al.^1^ (2015) demonstrated that single-session application immediately after surgical tooth extraction was more effective on pain, edema, and trismus as compared with late applications.

A control group was not formed in this study, which can be considered a limitation. The literature comprises many studies comparing PBM with a control group.^8 , 12 , 13 , 23 , 32^ Although some studies are reporting no effect of PBM as compared with a control group, the majority have reported positive effects of PBM.^8 , 12 , 13 , 23 , 32^ Accordingly, this study was planned based on the theory that PBM is more effective as compared with a control group and was designed to evaluate the effectiveness of different wavelengths and number of sessions without including a control group.

## Conclusions

Among the study limitations, after dental surgery, an immediate single-session of PBM is a preferable option since it provides similar outcomes to those obtained by repeated applications within 24 hours. Moreover, according to the results of this study, we suggest that the effects of laser applications at 810 nm and 940 nm did not significantly differ in PBM in terms of reducing or preventing postoperative complications (pain, swelling, and trismus) after removal of third molars with single or repeated sessions and by a point or regional applications. As laser devices operating at high wavelengths have higher costs, we considered that a single session of PBM with the most affordable laser device that has a wavelength within the therapeutic range could be beneficial for the patients in preventing postoperative complications. Accordingly, the disadvantages related to PBM, which is laser costs and time loss for patients and clinicians, could be minimized.
